# Racialization of public discourse: portrayal of Islam and Muslims

**DOI:** 10.1016/j.heliyon.2022.e12211

**Published:** 2022-12-10

**Authors:** Muhammad Kamran Sufi, Musarat Yasmin

**Affiliations:** University of Gujrat, Pakistan

**Keywords:** Islamophobia studies, Islamophobic discourse, Critical discourse analysis, Identity crisis, Global peace

## Abstract

Many developed countries like the USA, UK, Canada and European countries have diverse communities, including Muslim community outreach, as a result of immigration turning the world into a global village for all religions. Persecuting any one religion can lead to unrest and damage the calm of the society at large. This study critically examines the trends and research findings on Islamophobic discourse from 2001 to 2022 by investigating how linguistic strategies have been employed to present Muslims and Islam, the racialization of Muslims, the sense of identity crisis, and the way Muslims encounter and resist Islamophobia. An empirical study is also conducted to analyze the media discourse on recent incidents in Canada and New Zealand. For this purpose, 56 research articles are retrieved from the databases of four publishers: Taylor & Francis, Sage Publication, Pluto Journals, and Science Direct. A systematic review methodology and content analysis of the elected articles reveals that qualitative methodology was used in most articles and the UK and the US are the focal countries where most of the Islamophobia studies are carried out. Interviews and print media are found to be the preferred data samples for Islamophobia research. The most common theme in the articles is how anti-Muslim ideology was constructed by painting negative images of Muslims and Islam and subsequently presenting them as 'others.' The multiple effects of Islamophobia was paid considerable attention by the researchers of the reviewed articles. Themes receiving less attention are Islamophobia for political gain, identity crises, and the racialization of Muslims, whereas the factors behind Islamophobia received scant attention. The development of Islamophobia as a topic within the field of critical discourse analysis has been understudied. A critical discourse analysis of two recent incidents in Canada (2021) and New Zealand (2019) shows how various linguistic strategies are employed to construct negative images of Muslims and Islam.

## 1 Introduction

The modern world equipped with the advanced communication means of electronic media has become a ‘global village’ where all countries are tightly linked with each other. “A revolution in information, communications, transport and weapon technology has reduced the transaction costs of interaction over space and linked the world to the point where it has shrunk or even collapsed distance” (Porter, 2015). The global village's powerbase is the atomic bomb, reducing the likelihood of global war because of the widespead presence of atomic deterrents. At the same time, according to Porter (ibid), the global village is a more dangerous place where even superpowers like America and its allies live in terror in the wake of the 9/11 attacks (2001), 7/7 London bombing (2005), Paris attacks (2015) etc. Porter (ibid) asserts that fear persists in this small fragile world with the unprecedented condition of vulnerability and “aggressors can apply violence over large spaces” (Porter, 2015). Inequality reinforced by globalization and religious dissonance may also lead the world to become less peaceful and more dangerous.

The essence of all religions is peace and harmony, but the hardline religious scholars whether Muslim, Christian, Hindu, Jew, Buddhist spread hatred against other religions and impose their orthodox views to claim supremacy. Thus, religiously legitimized extremist movements in the name of Judaism (Jewish Zionists), Christianity (Christian Violent Fundamentalism, Christian Identity), Islam (Islamic State of Iraq and Syria, Al Qaeda), Buddhism (Bodu Bala Sena), Hinduism (Rashtriya Swayamsevak Sangh) etc. under the supervision of hardline religious scholars can instigate violence against other religions to fulfil their political objectives, threatening world peace (Cesari, 2015).

Islamophobia has been existent in the western countries from the time when Muslim and European Christians confronted each other during the Crusades in Asia Minor in 11th century (Bordbar et al., 2020). However, since the 9/11 attacks (2001), Islamophobia has intensified and become a serious problem. The mass media has the power to influence minds (Yasmin et al., 2018, 2019) worsened the situation by portraying undesirable, stereotypical, one-sided images of Muslims and Islam, so shaping public opinion against Muslims and Islam and causing fear, anxiety, and unrest among the common people, subsequently developing prejudice, racism, and conflict in society, leading to an unhealthy environment and disturbed peace in many European countries. Strabac and Listhaug (2008) analyzed negative attitudes in 13 European countries and found that Muslims faced negative attitudes significantly more than other migrant groups. Muslims and their religion, Islam, have been the constant target of hatred and prejudice in the west, which may be linked to the increase in Islamophobic hate crimes and violent acts against Muslims. Islamophobic attacks in one nation disrupt the peace of the whole world, especially the diverse communities with their globalized nature. It is time for the social scientists and policymakers of non-Muslim nations to investigate the dynamics of Islamophobia and its harmful effects to save their societies. They can play a positive role – with the assistance of the media – to reduce conflicts in communities based on religion and help bring harmony across the globe.

The development of Islamophobia studies in the field of critical discourse analysis is under investigated. There is a need to review the available literature and find out how Islamic discourse has been manipulated to construct anti-Muslim narratives, used to otherize and racialize Muslims, exploited for political gain, the effect on Muslim identity, and how Muslims resist Islamophobia. The current study focuses on the trends and findings of Islamophobic discourse studies to observe their growth within the boundaries of linguistics. The paper also presents a critical discourse analysis of two recent incidents in Canada (2021) and New Zealand (2019) to show how various linguistic strategies were employed to portray Muslims and Islam. The following three research questions were formed:

i. What are the trends and findings from some discourse studies on Islamophobia?
ii. How does the discourse about Islam construct anti-Muslim narratives to otherize and racialize Muslims?
iii. What linguistic strategies have been employed to portray Muslims and Islam in the media reports on Islamophobic incidents?

Islamophobia is hatred of Islam and Muslims. The Oxford English Dictionary defines Islamophobia as an “intense dislike or fear of Islam.” (Dictionary, 1989). According to Runnymede Trust's report (1997), it is “a shorthand way of referring to dread or hatred of Islam—and, therefore, the fear or dislike of all or most Muslims.” In 2017, the Runnymede Trust Institute gave a comprehensive and more extended definition of Islamophobia by associating it with racism as follows:

“any distinction, exclusion, or restriction towards, or preference against, Muslims (or those perceived to be Muslims) that has the purpose or effect of nullifying or impairing the recognition, enjoyment or exercise, on an equal footing, of human rights and fundamental freedoms in the political, economic, social, cultural or any other field of public life.” (Elahi and Khan, 2017).

In general, the discrimination and exploitation of Muslims in all spheres of life are considered to constitute Islamophobia.

**Figure 1:**
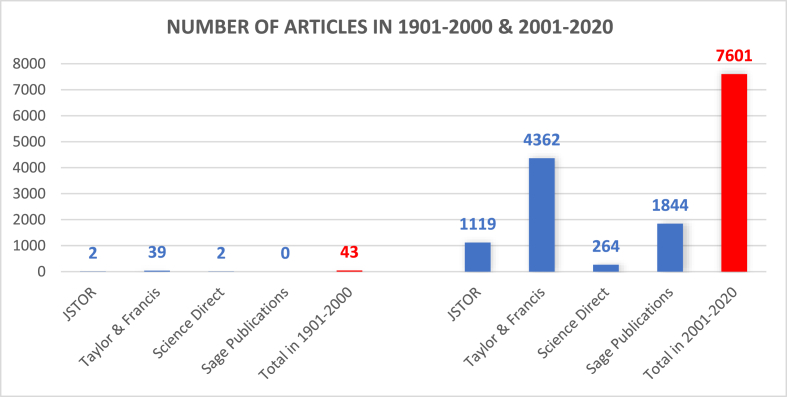
Number of articles yielded by search terms in 1901–2000 and 2001–2020.

Richardson (2012) traces out the history of Islamophobia and mentions that the term *Islamophobia* was derived from the French word ‘Islamophobie’, which Alain Quellien first used in 1910 to criticize the behaviour of French colonial administrators towards Muslim subjects. He further says that in English, Edward Said pioneered the use of Islamophobia in his article in 1985 to create a “connection … between Islamophobia and antisemitism” and criticized the writers who did not recognize “hostility to Islam in modern Christian West” as equal to antisemitism (Richardson, 2012). After the tragic event of 9/11, Islamophobia increased. However, the twentieth century showed minimal research work on Islamophobia compared to the first two decades of the twenty-first century. For example, from 1901 to 2000, the search for articles on the selected topics from the databases of four publishers, Taylor & Francis, Sage Publication, Pluto Journals, and Science Direct, yielded only 43, as illustrated in Figure 1. On the other hand, these same databases showed 7601 articles between 2001 and 2020, suggesting that 9/11 incident in 2001 became pivotal in increasing Islamophobia and attracting the attention of academic researchers.

## 2 Methodology

Following the guidelines and criteria set forth by the Preferred Reporting Items for Systematic Reviews and Meta-Analyses (Page et al., 2021), the researchers systematically conducted, on January 12, 2021, a review of the previous studies on Islamophobic discourse and the representation of Muslims and Islam to discover which studies needed to be investigated further. A systematic review is different from a literature review because it is a detailed and comprehensive plan of study to summarize the evidence surrounding particular questions (Tawfik et al., 2019) by following a precise procedure covering multiple databases (Robinson and Lowe, 2015). The databases of four publishers, Taylor & Francis, Sage Publication, Pluto Journals, and Science Direct, were selected to search for key terms: Islamophobia, Islamophobic discourse, critical analysis of Islamophobic discourse, its impacts, Islamophobic discourse othering Muslims, Islamophobic discourse and counter-narrative, and deconstruction of Islamophobic discourse. From the results returned, only research articles were considered in this study. Pluto Journals articles are on the JSTOR database from where Pluto Journals articles were taken.

### 2.1 The selection criteria were further narrowed down to (the criteria are illustrated in Figure 2, below)

A. Articles based on discourse analysis (DA) or critical discourse analysis (CDA) of Islamophobic text.

**Figure 2:**
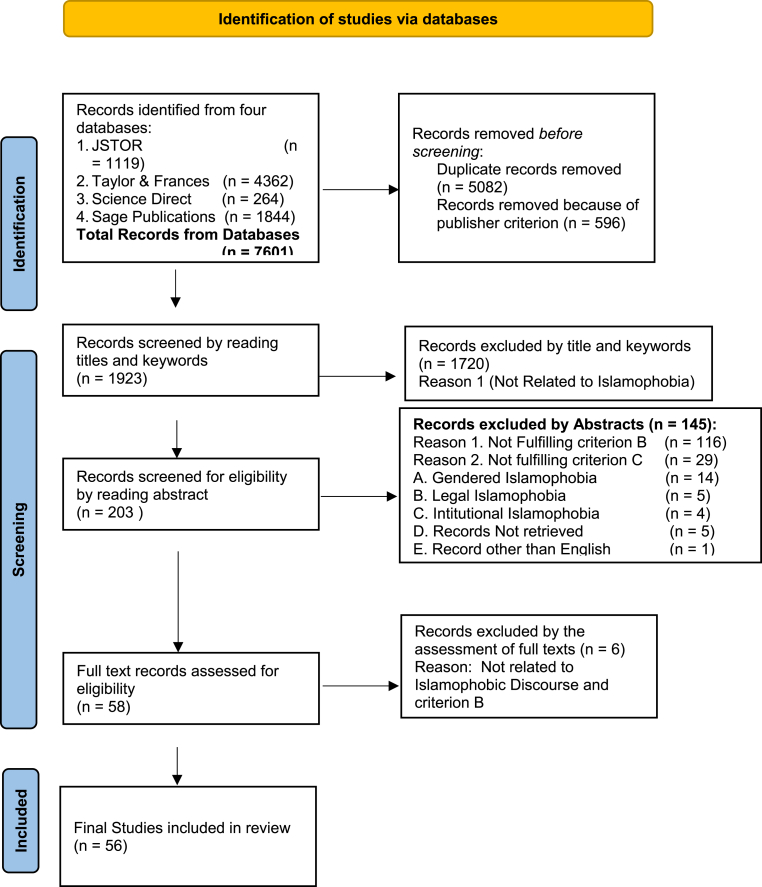
Diagram of Systematic review process (adapted from PRISMA, 2021; Page et al., 2021).
B. Articles based on at least one of the following themes: Islamophobia, Islamophobia and Muslims, Islamophobia and Counter-Narrative, Deconstruction of Islamophobic Discourse.
C. Other themes like gendered Islamophobia or legal Islamophobia or institutional Islamophobia were excluded from this research because of time and space restrictions.

The search terms from the databases of the four publishers yielded 7601 articles. Duplicate copies were removed (as illustrated in Figure 2) and following the publisher criteria, a further 596 articles were removed, leaving 1923 articles for screening for eligibility. After reading the titles and keywords carefully, 1720 articles were excluded because they were not related to Islamophobia, hence the sample size was reduced to 203. A careful examination of the abstracts further reduced the number of articles to 58, while after reading each of the full-length articles, six more were excluded since they did not fulfill selection criteria A and B, reducing the number of articles to 52. As the study progressed into 2022, four more articles, fulfilling the selection criteria, from 2021 and 2022 were added. Hence, the articles selected for review in this study are 56, as mentioned in Table 1 along with their distribution across publishers.

**Table 1:** Total number of selected articles to be reviewed.

Sr. No.	Publishers	Selected Articles on the basis of DA/CDA of Islamophobic text	Selected Articles on the basis onMajor Theme(s)	Selected Articles from 2021 & 2022	Total
1	Pluto Journals	3	7	0	10
2	Taylor & Francis	9	12	1	22
3	Science Direct	1	2	1	4
4	Sage Publication	7	11	2	20
	**Total**	**20**	**32**	**4**	**56**

### 2.2 Codification

The selected articles were coded as TF (Taylor and Francis), S (Sage Publication), P (Pluto Journals) and SD (Science Direct). A number is attached to each article according to the selection order in which they appeared in the databases, such as TF1, TF2, TF3, and so on.

### 2.3 Data analysis

Content analysis was employed to compare, contrast and categorize data following Ahmed and Matthes' (2017) proposed two-step analysis procedure, which provides the basis for quantitative analysis (dealing with RQ1) and qualitative analysis (dealing with RQ2). Content analysis is defined as “a family of research techniques for making systematic, credible, or valid and replicable inferences from texts and other forms of communication” (Drisko and Maschi, 2016). An initial table in Microsoft word was developed to record the analysis results incorporating the subcategories of the research questions (RQ1 & RQ2) from careful reading of each article - the title of the article, name (s) of the author(s) and journal in which the article was published, the objective and focus of the article, sample type, approach/strategy of inquiry, research methods, data collection tools, findings, publisher's name, year of publication, and place of research. The first research question (RQ1) and its subcategories deal with explicitly mentioned quantitative-based descriptive information concerning the trends in Islamophobic discourse studies. The second research question (RQ2) and its subcategories are related to the findings of Islamophobic discourse studies, including qualitative-based thematic information derived from reading the reviewed articles in detail. For the third research question (RQ3) following on from Systemic Functional Grammar (SFG) (Halliday et al., 2014), ‘nominal groups’ and ‘transitivity’ are taken into account for linguistic analysis under Van Dijk's Socio-Cognitive approach (2008, 2009).

## 3 Findings

### 3.1 Trends in Islamophobic discourse studies

The subcategory response to RQ1 include trends in Islamophobic discourse studies identified from the distributions of the reviewed articles by years, research methods used, countries in which the studies were conducted, methods of discourse analysis, linguistic or non-linguistic analysis, and sample types.

#### 3.1.1 Distribution of the reviewed articles by years

**Figure 3:**
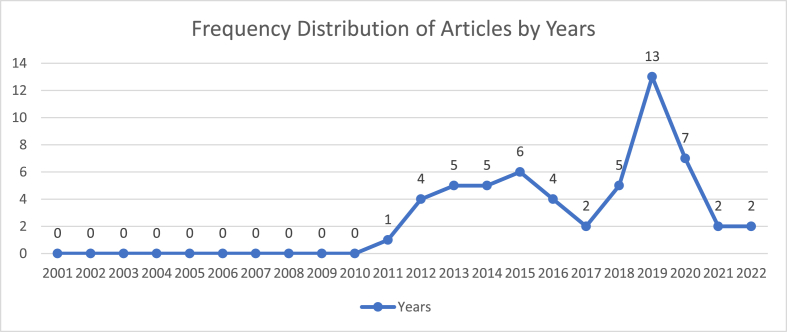
Frequency distribution of the reviewed articles by years.

The data showing the distribution of the reviewed articles by year is presented in Figure 3. Although a considerable number of research articles on Islam and Muslims were published in the first decade of the 21^st^ century, no article was found that fulfilled the selection criteria. In 2011, there was only one publication. There was a trend of gradual increase to 2015 with 6 publications, but a drop in the publication of relevant articles in 2016 and 2017, but an increase in 2019 and 2020. 2021 and 2022 each had 2 publications. A systematic analysis showed a gradual rise in researcher interest suggesting indirectly, a rise in Islamophobic events that could threaten global peace.

#### 3.1.2 Research methods used in previous research

**Figure 4:**
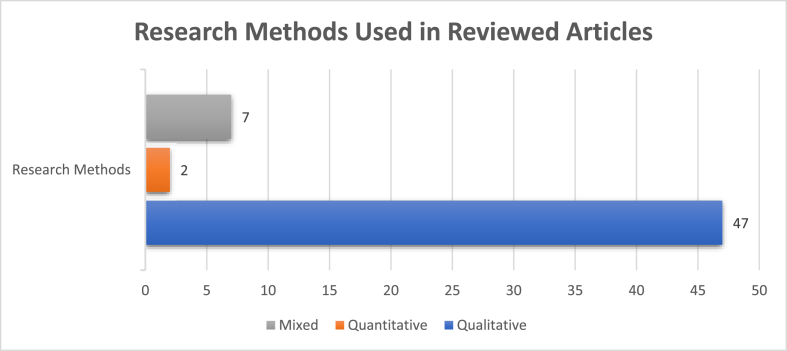
Research methods used in the articles reviewed.

The most adopted research method was the qualitative method, more than 6 times higher in number than the mixed method. The quantitative method was used only in two articles, as shown in Figure 4. The results showed that the qualitative method was considered ideal for an in-depth study and analyzing discursive practices. A rising interest in mixed methods shows that researchers also felt inclined to strengthen their studies by utilizing more than one methods.

#### 3.1.3 Countries of the reviewed articles

**Figure 5:**
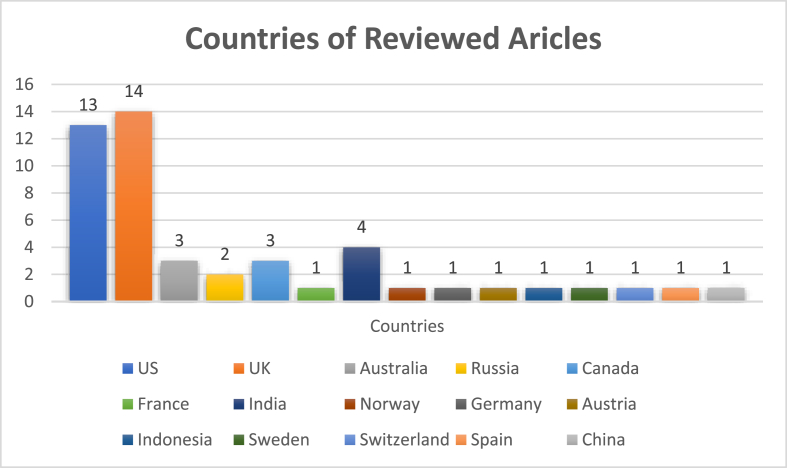
Countries of the articles reviewed.

The analysis revealed that most of the studies on Islamophobia were conducted and published in the United Kingdom and the United States, as presented in Figure 5. The subject was less explored in other European countries. France, Norway, Germany, Sweden, Switzerland, Spain and Austria had one study each out of the 56 articles. In China there was also one publication in the selected study area. Australia and Canada and Russia had 3, 3, and 2 publications, respectively. On the other hand, some significant work came from India with 4 studies. The results suggest, there may be a higher incidence of Islamophobia in US and UK leading to a higher awareness of the problem, which thus attracted the attention of researchers.

#### 3.1.4 Methods of discourse analysis adopted

**Figure 6:**
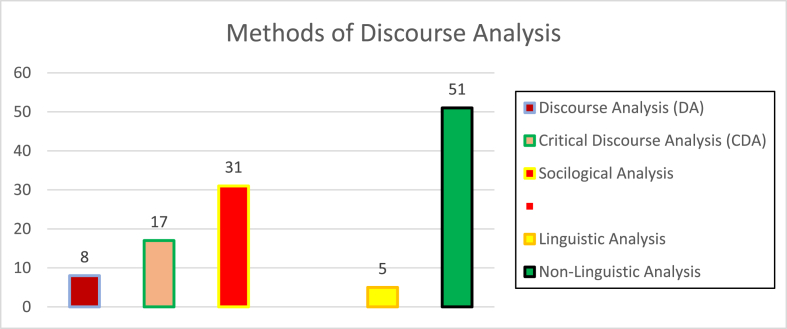
Methods of discourse analysis adopted in reviewed articles.

As shown in Figure 6, 31 of the reviewed articles, were based on sociological rather than linguistic methods. There was little linguistic analysis of the data in the reviewed articles. Of the 56 studies with linguistic analysis, CDA was the most favored method used.

These finding suggest there is great scope for language researchers – applied linguists to study the language used by the victims of Islamophobia, the perpetratos – including the media, and government official statements - to discover what lies behind their utterances.

#### 3.1.5 Types of samples used

**Figure 7:**
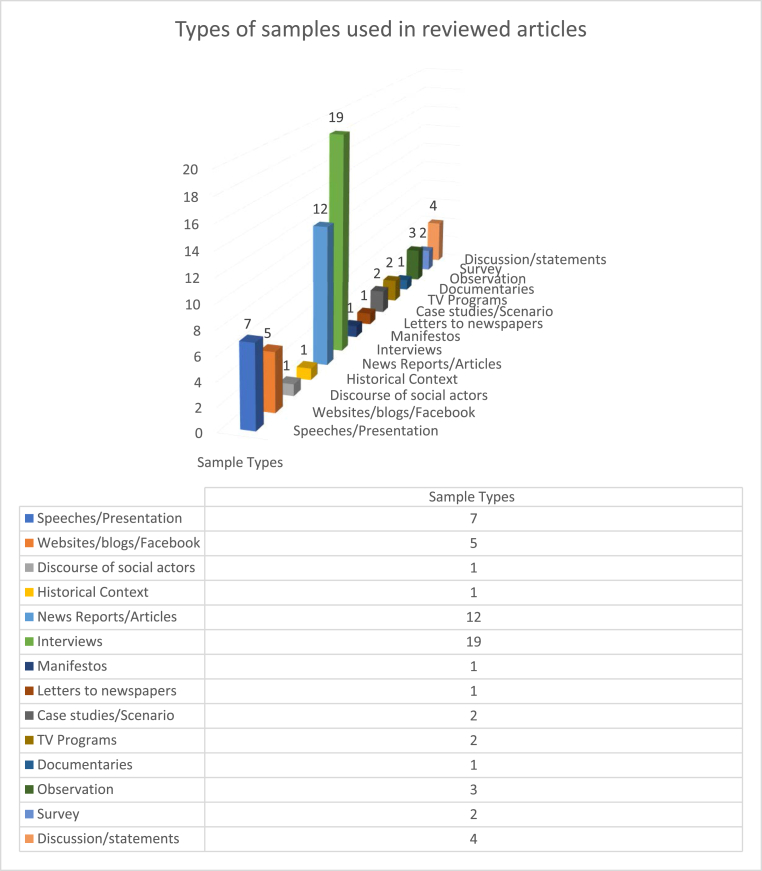
Sample Types used in reviewed articles.

As presented in Figure 7, 19 of the examined articles relied on interviews, and 12 on news reports/articles. 7 speeches/presentations were taken as samples. Two categories of samples, websites/blogs/web series/Facebook and discussion/debates/statements, were each used 5 times and observation samples 3 times. The selection of case studies/scenarios, TV Programs and Survey as sample types had 2 occurrences. Seven types of samples, the discourse of social actors, historical context, manifestos, letters to newspapers, questionnaires, tweets and documentaries, each category had one example. Overall, interviews and newspapers were the preferred sources of samples for researchers.

### 3.2 Major themes in Islamophobic discourse studies

After a careful reading of the articles, seven major themes emerged as the findings in response to RQ2, illustrated in Table 2. The emerging theme of anti-Muslim ideology/narratives by portraying Muslims as others had the highest occurrence of 23 times. In contrast, the least interesting themes for the researchers were the factors behind Islamophobia (3), Islamophobia and identity crisis (5), and Islamophobia for political gain (5). The second most common theme was related to the effects of Islamophobia, which was found for 13 times. The themes of encountering/resisting Islamophobia occorred 8 times and Islamophobia as racism for 7 times in the reviewed articles.

**Table 2:** Main Themes on Islamophobia in research articles.

Sr. No.	Theme	Total No. of Articles
1	Emerging anti-Muslim Ideology or narrative by portraying Muslims as others	23
2	Islamophobia as racism	7
3	Islamophobia and identity crisis	4
4	Effects of Islamophobia	13
5	Encountering/Resisting Islamophobia	8
6	Islamophobia used for political gain	5
7	Factors behind Islamophobia	3

#### 3.2.1 Emerging anti-Muslim ideology or narrative by portraying Muslims as others

Out of the 56 selected articles, 23 were related to the theme of emerging anti-Muslim ideology, focusing on investigating the negative representation of Islam and Muslims. Muslims were portrayed as a potential threat to the West, discriminated against and constructed as ‘others’ through certain discursive strategies such as self-other schemata, radical labeling, language games (Wittgenstein's DA methodology), word choices (metaphors, pronouns and other lexical choices), rhetorical devices, logic and argument, modality, idioms, clichés.

Following the self-other schema, Donald Trump presented Muslims in negative terms through radical labelling in his policy speech at the American Israel Public Affairs Committee (Khan et al., 2020). He described Muslims as the cause of suffering and destruction and projected himself as a hero and the savior of America. Mohiuddin (2019) claimed that the essentialism of Muslim culture prevents Muslims from being assimilated to Eurocentric culture. Without removing their differences they were “constructed as the West's antagonistic other”. This suggests the West wants Muslims to adapt to the cultural frame created by the West. Following on Norman Fairclough's (1995) critical discourse analysis theory, Ragozina (2020) analyzed linguistically the modality of ‘want’ and ‘can’ to unravel the way the image of Muslims was constructed in Russian national newspapers. Muslims were portrayed “as an alien culture excluded from Russian social and political space” (Ragozina, 2020). There, sharia law was present as in opposition to the law of state and disruptive of social harmony. Waikar (2018a) analyzed Trump's speeches and interviews and found that through claiming hegemonic neoliberalism of Islam and Muslims, Trump's goal was to “otherize” Islam and Muslims and to perpetuate this assigned status. He concluded that through narratives against Islam and Muslims, Trump tried to associate terrorism with radical Islam as a potential threat to the American security. Furthermore, Islam and Muslims were misrepresented in French newspapers as pointed out by Ait Abdeslam (2019). Islam was presented as a radical religion and the attitudes and beliefs of Muslims were considered incompatible with French culture. France aimed to control and moderate Islam to domesticate it and make it compatible with French culture. In another study, it was argued that the representation of Muslims and Irish communities in the British press constructed them ‘suspect’ (Nickels et al., 2012). Moreover, Kassimeris and Jackson (2012) critically analyzed correspondents' letters about building ‘Dudley Mosque’ published in UK newspapers, which revealed that Muslims were perceived as threat to the national and local culture of UK. Thus, they were inherently regarded as ‘others’. Similarly, Ali (2014) claimed that Muslim students were constructed in public discourse “as pre-modern figures” and “as an embodied physical threat.” (Ali, 2014). Bangstad and Helland (2019) demonstrated that Storhaug's writings on Islam and Muslims were made up of rhetorical devices to incite fear of Muslims by making them akin to Nazis. Another researcher analyzed media releases, press statements, press conferences, media interviews, media articles, and television programs as the backdrop to the ‘Sydney Siege’ and found that “Muslims are placed precariously at the national margin.” The ‘good ones’ were to be included and the ‘bad ones’ were to be excluded (Colic-Peisker et al., 2016). Al-Azami (2021) analyzed the language used in right-wing British newspapers - *The Sun, The Daily Mail*, and *The Daily Telegraph* - and found that Muslims and Islam were stigmatized, either overtly or covertly, and associated with terrorism.

Khan et al. (2021) critically analyzed the selected Islamophobic tweets of Trump, the former president of the USA, and found that Trump used language rhetorically to exclude people of different ethnic identities, especially Muslims, to appeal to rightwing voters and that creating *“us” vs. “them”* was the way to make “America Great Again”. Burke and Diba (2020) investigated the English Defence League's Facebook page that showed positive images of Jews in order to demonize Muslims as the ‘new Nazis’. Along with Islamic extremists, ordinary, everyday Muslims were also targeted. In another article, Sharifi et al. (2017) critically analyzed CNN's talk show, *Global Public Square* (GPS), and found that CNN was biased and stereotyped Muslims at all levels. Muslims were associated with “terrorism, injustice, backwardness, insecurity and alienation” (Sharifi et al., 2017). Another research articlehighlighted Muslims' experience with and responses to Islamophobia online and in face-to-face encounters in the US, and it was found that every Muslim internet user in the US had experienced hyper differential public encounters (Eckert et al., 2021). Moosavi (2015a) examined the speeches of British Cabinet ministers of the Labour Government (2001–2007) revealing that mainstream politicians of Labour Government stigmatized Islam and Muslims and did nothing to diminish Islamophobia. The findings showed, Moosavi claimed, that Muslims were considered outsiders and not respected as citizens, rather they were blamed as troublemakers and taken as opposed to Britishness.

Another analysis of the podcasts, interviews and speeches of Narendra Modi by Waikar (2018b) identified a subtle form of Islamophobia in narratives whereby Muslims were presented as subordinate to Hindus. Sumra (2020) investigated the Indian media and found Islam and Muslims were presented in a hostile manner as “socially backward, non-dynamic, responsible for wrong doings” (Sumra, 2020). Siyech and Narain (2018) due to slaughtering cows in India, – (Hindu ethics considers all animal life sacred, especially zebu cows and most Indians are vegetarian) Muslims were constructed others by extremist Hindu organizations such as RSS, VHP and BD to justify violence against them. An article by Acim (2019) found that Muslims were viewed as potential threats in United States and Europe. They were “the green menace” and they had replaced the “red menace” of communism (Acim, 2019).

Kunst et al. (2013) developed the Perceived Islamophobia Scale (PIS) to highlight the psychological stress Muslim minorities have faced in Europe found that Pakistanis perceived less Islamophobia in Britain compared to Turks and Arabs in Germany. Moreover, Maghrebis in France faced the highest level of Islamophobia of all Muslim minority groups in the study (Kunst et al., 2013). Törnberg and Törnberg (2016) critically analyzed the corpus of the Swedish internet forum, Flashback, and found that Muslims were presented as a homogenous outgroup involved in conflict, violence and extremism. They also discovered that the internet amplified the polarizing effects of Islamophobia. A recent study conducted by Navarro-Granados & Llorent-Bedmar (2022) in Spain found that the Muslim community felt discriminated against and unjustly treated.

#### 3.2.2 Islamophobia as racism

Although the word ‘racism’ was not included in the search terms in the databases, in the articles selected, the theme of racism against Islam and Muslims along with other themes appeared in six articles. The Runnymede Trust defines Islamophobia as follows: “Islamophobia is anti-Muslim racism,” (Elahi and Khan, 2017). Islamophobic web pages “create a seemingly mainstream political position by framing racist standpoints as a defence of Western values and freedom of speech” (Ekman, 2015). Brown and Saeed (2015) explored racialization of Muslim women studying in UK universities in general public sphere and found wearing the veil was viewed as a threat to the liberal tradition of British values. Muslim Women were racialized by wearing radical Muslim signs such as the veil.

Garner and Selod (2015) investigated the racialization of Muslims through Islamophobia and asserted that racism was fluid and not restricted to time and place. “Regardless of physical appearance, country of origin and economic situation”, all Muslims were perceived as a homogenous group, as a single race and demonized in Islamophobic discourse. Another researcher dealt with the racialization of Muslim converts in Britain. Muslim converts faced subtle Islamophobia as such their religious conversion was taken as racial conversion. Their religious conversion made them ‘others’ even though they had been the part of the white majority earlier (Moosavi, 2015b). Cheng (2015) investigated Swiss federal parliamentary debates and found a difference between Islamophobia and Muslimophobia. Unfounded hostility against Islam was Islamophobia whereas unfounded hostility against Muslims was Muslimophobia. In this context, while negative sentiments referring to Muslims were racist, while anti Islam sentiments could only be racist if Islam was used as metonymy for Muslims. Abbas (2020) pointed out that othering Muslims by a virulent strain of Islamophobia was a reinvention of racism.

#### 3.2.3 Islamophobia and identity crisis

The theme of identity crisis appeared in four articles. In general Muslims minority communities in Europe suffered from identity crisis when they were made ‘others’ through Islamophobic discourse, making them vulnerable in the Western society without the right to develop their own identities. The west wanted Islam and Muslims to adopt in a modified version according to Eurocentric ideology (Mohiuddin, 2019). Muslim students in US experienced loss of identity, as they were made to feel unable to “use rational logic, participate in liberal democratic society, or engage in a dialog to express disagreement” (Ali, 2014). Lynch (2013) highlighted the aftermath of 7/7 and subsequent related attacks, caused young Muslims in Britain to increasingly incorporate British identity at the loss of their own identity.

Abu-Ras et al. (2013) explored the effects of 9/11 on the memories and sense of identity of American Muslim physicians (AMPs) and found that AMPs initially experienced negative feelings about their identity when facing prejudice in US, but it helped them to develop their religious identity and they took adaptive measures.

#### 3.2.4 Effects of Islamophobia

As Islamophobia is a negative reaction, it has negative effects for both Muslims and non-Muslims. It can disrupt the peace of a community. It projects fear, anxiety, hatred and violence. Recent research suggests that fear created by counter-jihadist bloggers resulted in violence against Muslim minorities in Europe (Ekman, 2015). Bouma (2011) explored the strong anti-Muslim undercurrent of Islamophobia in Australia that affected the peaceful and productive cooperation among people belonging to different religions, making the way to world peace. Akbarzadeh observed the effects of Islamophobia on the Australian Muslim community and discovered that it weakened the bond between Muslim Youth and Australian society and disturbed the health of the environment (2016). Alizai (2020) investigated negative impacts of Islamophobia on Muslim students in Canadian universities. She found out that Muslim students suffered from the adverse effects of Islamophobia becoming isolated, ever conscious of surveillance, concerned about safety issues and facing unexpected violence. Moreover, Nickels et al. (2012) revealed the construction of a ‘suspect community’ through Islamophobia in the British media encouraged the violation of civil liberties by the state security apparatus.

An auto-ethnography inquiry conducted in UK, found that victims of Islamophobia suffered from “depression, sadness, fear, anxiety, suspicion, anger, helplessness and isolation” (Zempi and Awan, 2017). They had to bear physical, emotional and psychological impacts. In addition, Shaw (2012) critically analyzed the newspapers' coverage of the 7/7 London attacks and found that marginalization of Islam and Muslims in the media had negative implications for intercultural communication. It also affected peace journalism and human rights journalism. In their research article on the impact of Islamophobic hate crimes, Walters et al. (2020) discovered that Muslim victims of hate crimes developed a greater sense of ‘shared suffering’ with other group members compared to LGBT victims. Hate crimes gave rise “to anger-based emotions and anxiety-related emotions” (Walters et al., 2020). Another article found that the use of the term ‘radical Islam’ was associated with terrorism in the US media and increased a general fear of Islam and Muslims among people (Hoewe and Bowe, 2021). Furthermore, Pilkington and Acik (2020) found that lack of individual recognition and inequality of young Muslims created social activism among them. Lan and Navera's (2022) study in China revealed that social media discourse and government discourse frame Islam as incompatible with mainstream culture and the patriotism demanded for assimilation into the main culture.

Democratic processes in the Muslim world were suppressed because of Islamophobia in the US and Europe, according to Kaminski (2014). Another study showed that the continuous negative representation of Muslims in the media put pressure on them “to present themselves in non-threatenisng and welcoming ways to others” (Pihlaja and Thompson, 2017). Muslims felt discriminated against and confused about how to response in meaningful ways.

#### 3.2.5 Encountering/resisting Islamophobia

Islamophobia is the suppression and exploitation of Muslims. Being minority communities in the West, they are in a weak position. However, there are attempts to encounter Islamophobia and resist it. In eight articles out of the 56 identified articles, the theme of encountering/resisting Islamophobia appeared. Navarro-Granados and Llorent-Bedmar (2022) investigating in Spain, found Muslims had lower academic qualifications, and feelings of not belonging to Spain or their country of origin or that of their parents, meant they gave more importance to Islam in their lives. Wheatley (2019) pointed out that Bassim, a Muslim student in United States, resisted Islamophobia by writing poetry in English as a subtle way of releasing religious emotions through poetry and combating Islamophobia. Moreover, Smith (2020) highlighted the role of non-Muslim social workers cooperating with Muslims in Canada. It was suggested that the principles of “critical reflective, person-centered, and social justice practices” adopted by non-Muslim social workers could help Muslims who felt the impact of Islamophobia. Another research article by Lathion (2015) on strategies to resist Islamophobia in Europe suggested a disturbing religious situation could be managed by a secular legal framework, but that repressive policies were unworkable for stopping acts of violence. Muslims need their self-confidence restored so that they can easily criticize and dismiss statements threatening them. Hammad (2020) in article investigated how “Guess Who's Muslim” used humor as a satirical technique to challenge Islamophobia. “Guess Who's Muslim” was a web series played on YouTube channel, *West Dawn Media*, to counteract Islamophobia. Hammad (2020) believed that the internet was an alternative media forum where Muslims could represent themselves. However, he was of the view that humor can be a subtle with more than one interpretation. People might understand humor according to their own background knowledge and cultural practices. It was also possible that the humor created to challenge Islamophobia might reinforce the very stereotypes regarding Islam and Muslims.

Anderson (2015) focused on the need for a ‘peace journalism’ approach to counteract Islamophobia and that standards for reporting issues relating to Islam and Muslims could curtail Islamophobia. Further, the self-representation of ‘mainstream Muslim leaders’ could provide an opportunity for Muslims to “negotiate and perform politics of belonging and inclusion in United States” and resist Islamophobia according to McGinty (2012). Beshara (2019) claimed that Muslims in United States used epistemic and ontic methods to resist Islamophobia and believed that these methods of resistance were the ‘actual liberation’.

#### 3.2.6 Islamophobia for political gain

Islamophobia is a political term exploited by populist political parties for political objectives. Politicians used Islamophobic discourse to polarize the electorate and gain a victory. They also used it to hide their own follies and mistakes and blame Muslims for all their problems. Kaya and Tecmen (2019) found that European populist parties, Alternative for Germany (AfD) in Germany, Front National (FN) in France, Party for Freedom (PVV) in the Netherlands, Five Star Movement (M5S) in Italy, and Golden Dawn (GD) in (Greece), exploited fear of Islam as a political tool to motivate their supporters and to project themselves. The researchers maintained that the ruling political groups in Europe constructed Islamophobia as an ideology to cover up their own failure and gain control over social, economic, political and legal forces.

Stein and Salime (2015) analyzed anti-Muslim pseudo-documentaries and found these videos of fear created political polarization and right wing parties used them as a political weapon. These videos were also used to justify US interventions in the name of War on Terror. Abbas (2020) claimed the rise of Islamophobia by the UK political media had the objective of diverting people's attention from grave issues such as inequality, poverty and uneven economic conditions. A research article by Trein (2018) analyzed the debates on Islam in Germany and found that the rationale behind Islamophobia was to “establish political and religious subjectivities” and to ensure the “governability of Muslims” (Trein, 2018). Hafez (2019) compared the rise of antisemitism in 1876–1934 and Islamophobia today and found that Islamophobia served the political objectives of the rightwing populist FPO against the Social Democrats in Austria.

#### 3.2.7 Factors behind Islamophobia

Only 3 of the 56 reviewed articles included the factors behind Islamophobia. Ogan et al. (2014) found that in Europe and US, political conservatism was the main reason behind negative attitudes to Muslims and Islam. Shukri (2019) found that in Indonesia, Islamophobia was caused by rising Islamist extremism in that country. MacDonald (2015) demonstrated that misinformation about Islam was one of the crucial factors for the development of Islamophobic views.

## 4 Discussion

This study is a systematic review through content analysis of the trends and findings in 56 academic studies on Islamophobia retrieved from the databases of four publishers, Taylor & Francis, Sage Publication, Pluto Journals, and Science Direct. The analysis revealed that the start of the twenty first century showed a considerable rise in the number of academic studies on Islamophobia. Perhaps the tragic incident of 9/11 in 2001 and later terrorist attacks openly associated with Muslims prompted reactions in the media and attracted the attention of academics. Another reason for Islamophobia might be pressure from the economic recession 2007–2009 in Western countries, which might have considered Muslim immigrants a burden to their economy. The second decade of the twenty first century saw the incorporation of discourse analysis (DA) and critical discourse analysis (CDA) in Islamophobia studies suggesting linguists started to take interest in Islamophobic discourse. However, most articles that used DA or CDA methods for data analysis did not follow a specific DA or CDA model, possibly due to early stages of DA and CDA in Islamophobia studies. There is still a need to analyze Islamophobic discourse rigorously and critically in different contexts. Linguistic analysis of the data used in few of the articles identified for analysis, again, without following a prescribed model, perhaps because Islamophobic discourse has only recently been subject to linguistic analysis. Only in two studies Wittgenstein's approach of language games was exploited. The analysis further revealed that the most commonly used research method in the reviewed articles was qualitative. The reason might be the subject, Islamophobia studies, mostly involve negative discourse, attitudes and behaviors against Muslims and Islam, where the data is text/discourse not numerical. In the examined articles, the UK and the US carried out the majority (as illustrated in Figure 5) of the Islamophobia studies possibly because Islamophobia increased as a reaction to the collapse of US World Trade Centre in 9/11 attack and the UK 7/7 London attacks. These two countries were at the center of the production of anti-Muslim narratives, Islamophobia studies were conducted there. France was also directly hit by the 2015 Paris attacks but quite surprisingly, there was only one study carried out in France in the selected articles. The analysis revealed that interviews were the most common form of data source adopted by researchers, possibly because interviews provide in-depth insights into the interviewees concerning their experiences, attitudes, beliefs and opinions. Newspaper data was the second most popular data source because of its easy access.

The findings from the articles showed researchers were more interested in the way anti-Muslim ideology and narratives were constructed through the discursive representation of Muslims and Islam targeting Muslims representing them as external ‘others’, which made their views racially based. Although there were three studies on the Islamophobic discourse of Donald Trump's (former US President) anti-Muslim ideology, they lacked rigorous linguistic analysis. Moreover, in another study of Narendra Modi's (Indian Prime Minister) narratives against Muslims and Islam, there was no linguistic analysis. Other political heads like Emmanuel Macron (French President) and Boris Johnson (UK Prime Minister) used Islamophobic discourse. The attitudes of political heads of powerful countries as revealed in their speeches and narratives are significant because of their they large scale influence. Therefore, there should be comparative research of the Islamophobic narratives of the political heads of these countries.

In addition, the researchers in the selected articles were interested in the effects of Islamophobia, discovered to be a threat to a healthy and peaceful society. There were devastating effects on the Muslims in Muslim minority countries, who felt isolated and in constant danger of being victims of Islamophobia and experienced identity crises or were directly or indirectly hit by psychological trauma. There was only one study on Islamophobia in a Muslim majority country, Indonesia. Islamophobia studies in Muslim majority countries were understudied. It might be presumed that Muslims being in Muslim majority country would not be affected by Islamophobia. However, there could be a variant form of Islamophobia in Muslim majority countries, which need investigation. Furthermore, to combat Islamophobia, a few studies of the efforts of Muslims living in non-Muslim societies were carried out. The role of Muslims from Muslim countries and Muslim organizations in combatting Islamophobia was absent from the research. Curtailing the impact of any bad happening requires investigating its causes like a diagnostic process. The factors behind Islamophobia why it happens was not covered in the research articles.

Research into the representation of Muslims as victims and their perpetrators in media is lacking. The following section provides critical analysis of media reporting on the representation of Muslim victims and their perpetrators.

### 4.1 Case studies: Canada Shooting (2021) and Christchurch Mosque Shooting (2019)

Islamophobia has become a significant problem for most non-Muslim countries and has caused horrific events from vandalism to the killing of Muslims such as the recent Muslim family murder in Canada in 2021 and Christchurch mosque shooting in New Zealand in 2019. The reporting of these incidents in Canada's most popular newspaper, the Globe and Mail (Solutions, 2020), and New Zealand's most popular newspaper, the New Zealand Herald (Newspapers, 2019) is critically analyzed here to explore the way Muslim victims and their assailants are represented. For this purpose, ‘nominal groups’ and ‘transitivity’ from Systemic Functional Grammar (Halliday et al., 2014), are the frames for linguistic analysis within Van Dijk's Socio-Cognitive approach (2008, 2009). Only one news story from each newspaper has been selected for analysis because of time and space restriction. ‘According to Van Dijk (2001), critical discourse analysis (CDA) has a micro and a macro level. The micro level deals with the textual analysis of language use, discourse, verbal interaction and communication, while the macro level is concerned with socio-cultural practices like prejudice, discrimination, dominance, inequality, and the production and resistance of power from ideological and societal motivation. CDA bridges the gap between micro level analysis and macro level analysis with a cognitive interface.

#### 4.1.1 Critical analysis of Muslim family murder in Canada in 2021

In the aftermath of an attack on a Muslim family in London, the Ontario killing of four members and seriously injuring one, the news story, on June 8, 2021 in the Globe and Mail (Denette, 2021), did not contain a single word in the ‘nominal groups’ to represent the victim family's religious background, Islam, except in the headline, which follows as: “Attack on Muslim family in London, Ont.: What we know so far about the killings and suspect”.

In linguistic analysis of a nominal group ‘four family members’, ‘members’ is the head noun modified by a numeral ‘four’ and a classifier adjective ‘family’. Instead of ‘four family members’, there could be ‘four Muslim family members’ or ‘four members of a Muslim family’ to give a proper coverage to the incident as the investigating police officer, Waight, reported the “victims were targeted because of their Islamic faith” (Yancey-Bragg, 2021) and Prime Minister Justin Trudeau called it a ‘terrorist attack’. Twice the victims were described as a ‘Pakistani-Canadian family’ in which the ‘Pakistani-Canadian’ classifier indicates they were immigrants not pure Canadians. A nominal group, ‘a fifth’, who was not modified by any epithet, classifier or qualifier was an innocent and unfortunate boy of 9, became the recipient unearned suffering from an Islamophobic attacker, lost his whole family and was lying injured in hospital. Other nominal groups used for the victim family were: ‘four members of the Afzaal family spanning three generations’, ‘five family members’, ‘husband and wife Salman Afzaal 46 and Madiha 44’, ‘their daughter Yumna Salman 15’, ‘Mr. Afzaal's mother 74′, ‘the survivor’, and ‘the couple's nine year old son’. There is no mentioning of Islam or Muslims in these nominal groups which implies the disassociation of victims from their religion, Islam, might be the newspaper's effort to reduce the impact of Islamophobia. In addition, more space was used to describe the victim family compared to the perpetrator who was referred to by possible explanatory nominal groups such as: ‘a man’, ‘the suspect’, ‘the driver’, ‘the man’, ‘the truck driver’, ‘Nathaniel 20’. Only once was a classifier ‘truck’ used with ‘driver’ for perpetrator and the naming words used for him were also neutral. From this, it seems that the newspaper made little effort to expose the identity of the killer. Words like *killer, murderer, terrorist*, or *Islamophobic attacker* were not associated with him neither were his religion and color highlighted.

Analyzing transitivity in the excerpt: “Four family members who were out for a walk are dead, and a fifth in the hospital after a man drove into them deliberately” reveals, the following points. The relational clause ‘are dead’ is attributed to the carrier ‘four family members’ through an intensive process of transitivity and ‘a fifth’, another carrier's attribute is marked by circumstantial process of transitivity ‘in the hospital’ referring to his location. To reduce the force of agency and the cause of death, the agent of their dead is described anonymously later, using the indefinite article ‘a’, as ‘a man drove into them’ and instead of noun, the deictic ‘them’ was used as the goal, to refer to the for victim family. This suggests that the newspaper representation of the perpetrator was vague, perhaps deliberately so, possibly for legal reasons associated with the criminal justice system or not publicize the Muslim connection for fear of and Islamophobic issues or fallout.

#### 4.1.2 Critical analysis of Christchurch Mosque Shooting in New Zealand in 2019

The Christchurch mosque shooting in 2019 is one of the worst examples of Islamophobic attack, when 49 Muslims were killed and 80 others injured by a single attacker with a gun in a live streamed video. The news story was published in the New Zealand Herald (Herald, 2019) on March 16, 2019, one day after the incident. The ‘nominal groups’ used to represent the Muslim victims in the selected news story lacked any reference to Islam and Muslims as follows: “A gunman involved in the shootings, which left at least 49 people dead, livestreamed the attack in a chilling 17 min video”. In the nominal group ‘49 people’, ‘49’ is numeral used for the head noun ‘people’ a common noun without reference or connotation linking it to Islam or Muslim. In the nominal group ‘worshipper at the Al Noor Mosque’, ‘Al Noor Mosque’ as the qualifier used for ‘worshipper’ is implicit Islamic reference compared to Islam or Muslims. On the other hand, the perpetrator was described nominally as ‘a gunman’ and ‘the gunman’ classifier or qualifier words like *killer, murderer* or *terrorist* were not associated with him. Analyzing transitivity in the excerpt: “A gunman involved in the shootings, which left at least 49 people dead, live streamed the attack in a chilling 17 min video”, reveals that ‘49 people’ were not described as the direct goal of the actor gunman, but of the shooting. The deictic ‘which’ is used for an embedded clause as in ‘shooting, which left at least 49 people dead’, a language strategy to suppress the agency of the actor/agent.

Thus, it appears that ‘the Globe and Mail’ and ‘the New Zealand Herald’ used language strategies to avoid presenting attacks on Muslims as Islamophobic and hesitated to use words like Islamic or Muslim to refer to the victims. Moreover, both tried to hide the religious and ethnic identity of the perpetrators, used ‘soft’ references to them and suppressed their agency, suggesting that elements of western media are biased in representing anti-Muslim perpetrators and have not recognized Islamophobia and Islamophobic violence/terrorism yet. Unless or until Islamophobia is recognized by the general public through the media, better policies cannot be developed to curb it and to ensure peace in society the media can and should have a positive role to play.

## 5 Conclusion

This paper reviewed research articles concerning the rise in the alarming issue of Islamophobia. It reviews the association of Islamophobia with distancing or othering, racism, identity crises and political agendas. It further reviews the previous research on the factors in and effects of Islamophobia and resisting discourses and critically analysed the media reporting of two Islamophobic incidents and reveals that the religious identities of Muslim victims' were deliberately hidden whereas the Islamophibic attackers’ agency and identity were not revealed. This is an important study because it provides an essential guide for researchers of Islamophobia studies and (critical) discourse analysts. It points out the research gaps and opens the door for researchers to consider new themes to incorporate into Islamophobia studies. Finally, based on the trends and findings, the study offers the following suggestions:

• Studies on critical analysis of Islamophobic discourse should be carried out rigorously with specific CDA approaches such as the ‘socio-cognitive approach’ (Van Dijk, 1993, 2008), ‘Fairclough's (1995) text oriented form of discourse analysis (TODA)’ (Jørgensen and Phillips, 2002), the ‘discourse-historical approach’ (Wodak, 2001), ‘social semiotic framework for critical discourse analysis’ (Van Leeuwen, 2005) etc.
• The deconstruction of Islamophobic discourse was not used in any of the reviewed articles. Research on the deconstruction of Islamophobic discourse would be useful for encountering and resisting Islamophobia.
• The overall analysis of the reviewed articles found linguistic orientation in the Islamophobia studies was weak. There should be critical studies of Islamophobic discourse enriched with linguistic analysis following a specific procedure such as Systemic Functional Grammar (SFG) presented by Halliday et al. (2014) in his book, An Introduction to Functional Grammar.
• Studies of Islamophobia in Muslim countries were almost non-existent. Only one article out of 52 was found covering a Muslim country, Indonesia where one group of Muslims suffered from Islamophobia from another group of Muslims. Thus, Islamophobia studies should be conducted in countries where Muslims are the majority to observe the ‘insider’ reflections of Islamophobia.
• Comparative research should be conducted to critically analyze the Islamophobic discourse of the political heads of countries.
• Factors behind Islamophobia should also be studied further.
• In the reviewed articles, there were a few indications of the West's desire to mold Islam and Muslims according to Western values. This phenomenon is called the domestication of Islam and Muslims in the West, which requires a comprehensive research.
• Victimization of Muslims in the name of liberal democratic ideals and human rights is a topic, which should also receive the attention of the researchers.
• Forms of Islamophobia may differ from country to country and place to place. In the selected articles, one study compared the forms of Islamophobia and its practices in UK, Germany and France and distinguished different kinds of Islamophobia. It is suggested that more comparative studies could discover the nuances of Islamophobia in different countries.
• OIC, one of the biggest international organizations of Muslims can play a vital role in combatting and resisting Islamophobia. Comprehensive research is required to assess the role of OIC and other international organizations in curbing Islamophobia.
• The Role of powerful Muslim countries and their leaders should also be researched to counter Islamophobia
• Research should also be conducted in how Islamophobia and Islamophobic discourse could affect international relations.

## Declarations

### Author contribution statement

Musarat Yasmin:Conceived and designed the experiments; Analyzed and interpreted the data; Wrote the paper.

Muhammad Kamran: Conceived and designed the experiments; Performed the experiments; Analyzed and interpreted the data; Contributed reagents, materials, analysis tools or data; Wrote the paper.

### Funding statement

This research did not receive any specific grant from funding agencies in the public, commercial, or not-for-profit sectors.

### Data availability statement

No data was used for the research described in the article.

### Declaration of interest's statement

The authors declare no competing interests.

### Additional information

No additional information is available for this paper.

## References

[bib1] Abbas T. (2020). Islamophobia as racialised biopolitics in the United Kingdom. Philos. Soc. Critic..

[bib2] Abu-Ras W., Senzai F., Laird L. (2013). American Muslim physicians’ experiences since 9/11: cultural Trauma and the formation of Islamic identity. Traumatology.

[bib3] Acim R. (2019). Islamophobia, racism and the vilification of the Muslim Diaspora. Islamophobia Stud. J..

[bib4] Ahmed S., Matthes J. (2017). Media representation of Muslims and Islam from 2000 to 2015: a meta-analysis. Int. Commun. Gaz..

[bib5] Ait Abdeslam A. (2019). The representation of Islam and Muslims in French print media discourse: Le Monde and Le Figaro as case studies. J. Muslim Minority Affairs.

[bib6] Akbarzadeh S. (2016). The Muslim question in Australia: islamophobia and Muslim alienation. J. Muslim Minority Affairs..

[bib7] Al-Azami S. (2021). Language of islamophobia in right-wing British newspapers. J. Media Relig..

[bib8] Ali A.I. (2014). A threat enfleshed: Muslim college students situate their identities amidst portrayals of Muslim violence and terror. Int. J. Qual. Stud. Educ..

[bib9] Alizai H. (2020). Impact of Islamophobia on post-secondary Muslim students attending Ontario universities. Race Ethn. Educ..

[bib10] Anderson L. (2015). Countering Islamophobic media representations: the potential role of peace journalism. Global Media Commun..

[bib11] Bangstad S., Helland F. (2019). The rhetoric of Islamophobia: an analysis of the means of persuasion in Hege Storhaug’s writings on Islam and Muslims. Ethn. Racial Stud..

[bib12] Beshara R.K. (2019). From virtual Internment to actual liberation: the epistemic and ontic resistance of US Muslims to the ideology of (counter) terrorism–islamophobia/Islamophilia. Islamophobia Stud. J..

[bib13] Bordbar A., Mohammadi S., Parashi P., Butenko V. (2020). Globalization and islamophobia: critical view at globalization’s impact on expansion of islamophobia. J. Pol. L..

[bib14] Bouma G.D. (2011). Islamophobia as a constraint to world peace: the case of Australia. Islam Christian-Muslim Relat..

[bib15] Brown K.E., Saeed T. (2015). Radicalization and counter-radicalization at British universities: Muslim encounters and alternatives. Ethn. Racial Stud..

[bib16] Burke S., Diba P. (2020).

[bib17] Cesari J. (2015). Religion and politics: what does god have to do with it?. Religions.

[bib18] Cheng J.E. (2015). Islamophobia, Muslimophobia or racism? Parliamentary discourses on Islam and Muslims in debates on the minaret ban in Switzerland. Discourse Soc..

[bib19] Colic-Peisker V., Mikola M., Dekker K. (2016). A Multicultural nation and its (Muslim) other? Political leadership and media reporting in the wake of the ‘Sydney Siege. J. Intercult. Stud..

[bib20] Denette N. (2021). Attack on Muslim Family in London, Ont.: what We Know So Far about the Killings and Suspect. https://www.theglobeandmail.com/canada/article-london-ontario-anti-muslim-attack-victims-suspect-explainer/.

[bib21] Dictionary O.E. (1989).

[bib22] Drisko J.W., Maschi T. (2016).

[bib23] Eckert S., Metzger-Riftkin J., Kolhoff S., O’Shay-Wallace S. (2021). A hyper differential counterpublic: Muslim social media users and Islamophobia during the 2016 US presidential election. New Media Soc..

[bib24] Ekman M. (2015). Online Islamophobia and the politics of fear: manufacturing the green scare. Ethn. Racial Stud..

[bib25] Elahi F., Khan O. (2017).

[bib26] Fairclough N. (1995).

[bib27] Garner S., Selod S. (2015). The racialization of Muslims: empirical studies of islamophobia. Crit. Sociol..

[bib28] Hafez F. (2019). From “Jewification” to “Islamization”: anti-Semitism and islamophobia in Austrian politics then and now. ReOrient.

[bib29] Halliday M.A.K., Matthiessen C.M.I.M., Halliday M., Matthiessen C. (2014).

[bib30] Hammad O. (2020). North American Muslim Satire on YouTube: combatting or reinforcing stereotypes?. J. Media Relig..

[bib31] Herald N. (2019). Christchurch Mosque Shootings: Sky News Taken Down, Replaced with Fox Sports. https://www.nzherald.co.nz/nz/christchurch-mosque-shootings-sky-news-taken-down-replaced-with-fox-sports/SVUFMQVWZWA5Z7YJLUK2NFCZIU/.

[bib32] Hoewe J., Bowe B.J. (2021). Magic words or talking point? The framing of ‘radical Islam’ in news coverage and its effects. Journalism.

[bib33] Jørgensen M.W., Phillips L.J. (2002).

[bib34] Kaminski J. (2014). The Islamophobia industry, hate, and its impact on Muslim immigrants and OIC state development. Islamophobia Stud. J..

[bib35] Kassimeris G., Jackson L. (2012). British Muslims and the discourses of dysfunction: community cohesion and counterterrorism in the West Midlands. Crit. Stud. Terrorism.

[bib36] Kaya A., Tecmen A. (2019). Europe versus Islam?: right-wing populist discourse and the construction of a civilizational identity. Rev. Faith Int. Affairs.

[bib37] Khan M.H., Adnan H.M., Kaur S., Qazalbash F., Ismail I.N. (2020). A critical discourse analysis of anti-Muslim rhetoric in donald trump’s historic 2016 AIPAC policy speech. J. Muslim Minority Affairs.

[bib38] Khan M.H., Qazalbash F., Adnan H.M., Yaqin L.N., Khuhro R.A. (2021). Trump and Muslims: a critical discourse analysis of Islamophobic rhetoric in Donald Trump’s selected tweets. Sage Open.

[bib39] Kunst J.R., Sam D.L., Ulleberg P. (2013). Perceived islamophobia: scale development and validation. Int. J. Intercult. Relat..

[bib40] Lan T.X., Navera G.S. (2022). The slanted beam: a critical discourse analysis of anti-Islam and anti-Muslim discourse in China. Discourse Soc..

[bib41] Lathion S. (2015). Fight islamophobia in Europe? Less Islam and Muslims and more citizenship. Islam Christian-Muslim Relat..

[bib42] Lynch O. (2013). British Muslim youth: radicalisation, terrorism and the construction of the “other. Crit. Stud. Terrorism.

[bib43] Macdonald E.G. (2015). Muslims in Canada : collective identities. Attit. Otherment Can. Muslim Perspect. Radicalism.

[bib44] McGinty A.M. (2012). The “mainstream Muslim” opposing islamophobia: self-representations of American Muslims. Environ. Plann..

[bib45] Mohiuddin A. (2019). Islamophobia and the discursive reconstitution of religious imagination in Europe. J. Muslim Minority Affairs.

[bib46] Moosavi L. (2015). Orientalism at home: islamophobia in the representations of Islam and Muslims by the new Labour government. Ethnicities.

[bib47] Moosavi L. (2015). The racialization of Muslim converts in Britain and their experiences of islamophobia. Crit. Sociol..

[bib48] Navarro-Granados M., Llorent-Bedmar V. (2022). The Spanish Muslim community’s views on terrorism in the name of Islam and perceived discrimination: socio-educational measures. Int. J. Intercult. Relat..

[bib49] Newspapers (2019). Top Newspapers in New Zealand.

[bib50] Nickels H.C., Thomas L., Hickman M.J., Silvestri S. (2012). De/constructing “suspect” communities: a critical discourse analysis of British newspaper coverage of Irish and Muslim communities, 1974–2007. J. Stud..

[bib51] Ogan C., Willnat L., Pennington R., Bashir M. (2014). The rise of anti-Muslim prejudice: media and Islamophobia in Europe and the United States. Int. Commun. Gaz..

[bib52] Page M.J., McKenzie J.E., Bossuyt P.M., Boutron I., Hoffmann T.C., Mulrow C.D., Shamseer L., Tetzlaff J.M., Akl E.A., Brennan S.E. (2021). The PRISMA 2020 statement: an updated guideline for reporting systematic reviews. BMJ.

[bib53] Pihlaja S., Thompson N. (2017). I love the Queen”: positioning in young British Muslim discourse. Discour., Context Media.

[bib54] Pilkington H., Acik N. (2020). Not entitled to talk: (Mis)recognition, inequality and social activism of young Muslims. Sociology.

[bib55] Porter P. (2015).

[bib56] Ragozina S. (2020). Constructing the image of Islam in contemporary Russian print media: the language strategies and politics of misrepresentation. Relig. State Soc..

[bib57] Richardson R. (2012).

[bib58] Robinson P., Lowe J. (2015).

[bib59] Sharifi M., Ansari N., Asadollahzadeh M. (2017). A critical discourse analytic approach to discursive construction of Islam in Western talk shows: the case of CNN talk shows. Int. Commun. Gaz..

[bib60] Shaw I.S. (2012). Stereotypical representations of Muslims and Islam following the 7/7 London terror attacks: implications for intercultural communication and terrorism prevention. Int. Commun. Gaz..

[bib61] Shukri S.F.M. (2019). The perception of Indonesian youths toward islamophobia: an exploratory study. Islamophobia Stud. J..

[bib62] Siyech M.S., Narain A. (2018). Beef-related violence in India: an expression of Islamophobia. Islamophobia Stud. J..

[bib63] Smith S.J. (2020). Challenging Islamophobia in Canada: non-Muslim social workers as allies with the Muslim community. Journal of Religion and Spirituality in Social Work.

[bib64] Solutions A.P.R. (2020). Top 10 Canadian Newspapers. https://www.agilitypr.com/resources/top-media-outlets/top-10-canadian-print-outlets/.

[bib65] Stein A., Salime Z. (2015).

[bib66] Strabac Z., Listhaug O. (2008). Anti-Muslim prejudice in Europe: a multilevel analysis of survey data from 30 countries. Soc. Sci. Res..

[bib67] Sumra A. ul H. (2020). Muslims and Islam in Indian English press: exploring the Islamophobic discourse. Islamophobia Stud. J..

[bib68] Tawfik G.M., Dila K.A.S., Mohamed M.Y.F., Tam D.N.H., Kien N.D., Ahmed A.M., Huy N.T. (2019). A step by step guide for conducting a systematic review and meta-analysis with simulation data. Trop. Med. Health.

[bib69] Törnberg A., Törnberg P. (2016). Muslims in social media discourse: combining topic modeling and critical discourse analysis. Discour., Context Media.

[bib70] Trein L. (2018). Governing the fear of Islam: Thinking Islamophobia through the politics of secular affect in historical debate. ReOrient.

[bib71] Trust R. (1997).

[bib72] Van Dijk T.A. (1993). Principles of critical discourse analysis. Discourse Soc..

[bib73] Van Dijk T.A. (2001). The Handbook of Discourse Analysis.

[bib74] Van Dijk T.A. (2008).

[bib75] Van Dijk T.A. (2009).

[bib76] Van Leeuwen T. (2005).

[bib77] Waikar P. (2018). Reading islamophobia in hegemonic neoliberalism through a discourse analysis of donald trump’s narratives. J. Muslim Minority Aff..

[bib78] Waikar P. (2018). Reading islamophobia in hindutva: an analysis of Narendra Modi’s political discourse. Islamophobia Stud. J..

[bib79] Walters M.A., Paterson J.L., McDonnell L., Brown R. (2020). Group identity, empathy and shared suffering: understanding the ‘community’ impacts of anti-LGBT and Islamophobic hate crimes. Int. Rev. Vict..

[bib80] Wheatley L. (2019). ‘Quicksand of hate’: experiences of islamophobia and poetic resistance. Appl. Financ. Econ.: Stud. Cult. Educ..

[bib81] Wodak R. (2001). The discourse-historical approach. Methods Crit. Discour. Anal..

[bib82] Yancey-Bragg N. (2021). “Mass Murder”: Muslim family Targeted, Killed in Attack Motivated by Hate, Canadian Police Say. https://www.usatoday.com/story/news/world/2021/06/08/muslim-family-canada-killed-attack-motivated-hate-police-say/7598599002/.

[bib83] Yasmin M., Masso I.C., Bukhari N.H., Aboubakar M. (2019). Thespians in print: gender portrayal in Pakistani English print media. Cogent Arts Human..

[bib84] Yasmin M., Naseem F., Raza M.H. (2018). Creative marginalization of gender: a discourse analysis of advertisements in Pakistani newspapers. Creativity Stud..

[bib85] Zempi I., Awan I. (2017). Doing ‘dangerous’ autoethnography on Islamophobic victimization. Ethnography.

